# Arthropod Intelligence? The Case for *Portia*

**DOI:** 10.3389/fpsyg.2020.568049

**Published:** 2020-10-14

**Authors:** Fiona R. Cross, Georgina E. Carvell, Robert R. Jackson, Randolph C. Grace

**Affiliations:** ^1^ School of Biological Sciences, University of Canterbury, Christchurch, New Zealand; ^2^ International Centre of Insect Physiology and Ecology, Mbita Point, Kenya; ^3^ School of Psychology, Speech and Hearing, University of Canterbury, Christchurch, New Zealand

**Keywords:** arthropod, cognition, intelligence, problem solving, representation, spider

## Abstract

Macphail’s “null hypothesis,” that there are no differences in intelligence, qualitative, or quantitative, between non-human vertebrates has been controversial. This controversy can be useful if it encourages interest in acquiring a detailed understanding of how non-human animals express flexible problem-solving capacity (“intelligence”), but limiting the discussion to vertebrates is too arbitrary. As an example, we focus here on *Portia*, a spider with an especially intricate predatory strategy and a preference for other spiders as prey. We review research on pre-planned detours, expectancy violation, and a capacity to solve confinement problems where, in each of these three contexts, there is experimental evidence of innate cognitive capacities and reliance on internal representation. These cognitive capacities are related to, but not identical to, intelligence. When discussing intelligence, as when discussing cognition, it is more useful to envisage a continuum instead of something that is simply present or not; in other words, a continuum pertaining to flexible problem-solving capacity for “intelligence” and a continuum pertaining to reliance on internal representation for “cognition.” When envisaging a continuum pertaining to intelligence, Daniel Dennett’s notion of four Creatures (Darwinian, Skinnerian, Popperian, and Gregorian) is of interest, with the distinction between Skinnerian and Popperian Creatures being especially relevant when considering *Portia*. When we consider these distinctions, a case can be made for *Portia* being a Popperian Creature. Like Skinnerian Creatures, Popperian Creatures express flexible problem solving capacity, but the manner in which this capacity is expressed by Popperian Creatures is more distinctively cognitive.

## Introduction

For over a century, variation in human intelligence has been a topic of intensive study and debate (Wasserman, 2012) and, ever since Darwin, questions about the intelligence of non-human animals have also generated heated discussion and controversy. In an attempt to cast light on the evolution of intelligence, Macphail (1985, 1987; see also Macphail and Bolhuis, 2001) proposed what he called his “null hypothesis,” that there are no differences in intelligence, qualitative, or quantitative, between non-human vertebrates. On the basis of the evidence he considered, Macphail argued there was no compelling reason to reject this hypothesis because, as he saw it, reported differences between species on intelligence-related tasks could be attributed to “contextual variables.” In other words, he argued that if two species had been given the same problem to solve, but only one of these species succeeded or performed better than the other, this may have reflected a difference in intelligence, or it may have reflected an unrelated difference between the two species, such as in motivational factors, sensory systems, or other variables unrelated to intelligence (the “context”).

As illustrated by the commentaries accompanying Macphail (1987), this null hypothesis has been heavily criticized, with further reservations coming from subsequent findings. For example, non-human primates, but not pigeons, more rapidly solve one-dimensional, rule-based visual categorization tasks in which selective attention provides an advantage, compared with the two-dimensional integration tasks in which it does not (Smith et al., 2012). It is hard to see how contextual variables could account for this distinct difference between two species because, in these experiments, the same stimuli were used with each species and the main difference was only in the required responses, with monkeys having to touch one of two boxes on a screen and with pigeons having to peck one of two keys (Smith et al., 2010, 2011). As a more recent example pertaining to a similar category learning task, the performance of rats was intermediate to the performance of pigeons and non-human primates (Broschard et al., 2019).

On the whole, numerous reservations and rebuttals pertaining to Macphail’s null hypothesis seem valid, but there may be an indirect way in which this hypothesis has been useful because it encourages a comparative perspective and underscores the need to specify what “intelligence” means. Shortly before Macphail proposed his null hypothesis, Jensen (1980) had proposed a continuum of “intelligence,” where, at the bottom of this continuum, we find single-cell protozoans, before moving up to invertebrates, then up to lower vertebrates, then mammals, and finally reaching humans at the pinnacle (see Macphail, 1985). Macphail left invertebrates out when proposing his null hypothesis and this seems consistent with a widespread intuition (conventional wisdom) about invertebrates being limited to behavior that barely, if at all, qualifies as intelligent. We tend to associate intelligence with brains and there are often major differences in size between vertebrate and invertebrate brains. Octopuses may be an exception, but most invertebrates are arthropods (insects, spiders and their relatives) and it can easily seem a foregone conclusion that insects and spiders are just too small-brained to be intelligent. However, recent research on insects (Dyer, 2012; Giurfa, 2013, 2015) and spiders (e.g., Jakob et al., 2011) challenges the convention of assuming severe constraints on the expression of cognition by small animals. As cognition tends to be associated with intelligence, including arthropods can serve as a step toward taking a broader view of Macphail’s null hypothesis in terms of *scope* and *depth*.

Here, we will focus on *Portia*, a genus of jumping spiders (family Salticidae). These arthropods have unique, complex eyes and an exceptional ability for seeing detail in visual objects, making them especially suitable experimental subjects in research on behavior (Harland et al., 2012; Land and Nilsson, 2012), including behavior related to intelligence. With *Portia* in particular, we find flexibility and problem-solving capacities at a level that fits comfortably with the notion of what qualifies as “intelligence” when found in vertebrates. However, for a better focus, we might need at least a rough definition of what “intelligence” is.

For a definition, we can turn to Burkart et al. (2017, p. 2), who characterized non-human intelligence as an “individual’s ability to acquire new knowledge from interactions with the physical or social environment, use this knowledge to organize effective behavior in both familiar and novel contexts, and engage with and solve novel problems.” Their emphasis on flexibility and novelty highlights a key aspect of intelligence, this being that it applies to *domain-general* rather than *domain-specific* abilities.

When making comparisons in intelligence, Burkart et al. (2017) referred to between-species comparisons as differences in *G*, and within-species comparisons as differences in *g*. In this context, Macphail had mainly considered *G*, and an extension of his null hypothesis would predict that there are no differences in *g* as well as no differences in *G*. Yet, as Burkart et al. (2017) pointed out, there is considerable evidence from research on rodents (mice and rats) and non-human primates of differences in *g* and *G*. As an example of *g*, Matzel et al. (2003) tested 56 genetically-diverse mice in five different learning tasks (associative fear conditioning, operant avoidance, path integration, discrimination, and spatial navigation) and found that individual performances were positively correlated across tasks, with a single factor accounting for 38% of the total variance. As another example, Deaner et al. (2006) conducted a meta-analysis of non-human primate cognition studies using nine different experimental tasks, and found evidence for differences across genera, with great apes performing better than prosimians, New World monkeys, Old World monkeys, and lesser apes, suggesting differences in *G*.

Higher values of *G* have also been found to be correlated with larger brain size in non-human primates (Reader and Laland, 2002; Deaner et al., 2007) and, as Burkart et al. (2017) pointed out, this seems to present us with an evolutionary puzzle of general intelligence. It would seem that, when higher values of *G* evolve, we should find evidence of more domain-general intelligence compensating for the costs in resources and energy from growing bigger brains. Burkart et al. (2017, p. 20) refer to instances of domain-specific intelligence as “dedicated cognitive adaptations in response to recurrent fitness-relevant problems,” which seems to correspond at least roughly to the notion Fodor (1983) had of modular minds. Returning to Macphail (1987), the null hypothesis seems to imply that we should demand especially strong evidence before accepting conclusions about animals relying on domain-specific intelligence and, when this strong evidence is not delivered, that we should accept a null hypothesis of domain-general intelligence. Yet, when we consider the “puzzle” related to trade-offs (Burkart et al., 2017), maybe the null hypothesis should pertain to domain-specific, not domain-general, intelligence.

Compared with most vertebrates, salticids, like most arthropods, have brains that would comfortably fit on pinheads (Harland and Jackson, 2000). Yet, despite their tiny brains, salticids often display behavior that normally qualifies as “intelligent” when displayed by vertebrates. This makes it all the more important to understand how these abilities might have evolved.

As a step toward this goal, we will first address what we mean by “cognition” and “intelligence.” Next, we will review evidence for intelligent behavior in salticids, especially *Portia*, by focusing on experimental tasks involving pre-planned detours, expectancy violation, and novel problem solving. Based on the available evidence, we argue that *Portia* is an example of what Dennett (1996) called a Popperian Creature. Lastly, we consider the possible implications of research with arthropods for understanding the evolution of intelligence in non-human animals, and we discuss directions for future research.

## Intelligence on a Continuum

It may be a forlorn hope that any strict formal definition of “intelligence” will ever be widely accepted (Wasserman, 2012), but we should say something about the way we think of “intelligence” because, otherwise, we risk talking past each other. We will also discuss the distinction between intelligence and cognition, but we will begin here with Dennett’s (1995, 1996) informal notion of four Creatures (Darwinian, Skinnerian, Popperian, and Gregorian). When referring to these Creatures, we should acknowledge that we are doing a lot of simplifying because, with real organisms, we expect that the boundaries between Creature types will blur and that, when considering any one type, we can expect a continuum instead of a distinct category. As another simplification, we can envisage each of the four Creatures as having proficiency at responding to problems using solutions derived by trial-and-error.

A Darwinian Creature relies on a “hard-wired approach” (Geffner, 2013), with the animal’s “innate” or “instinctive” (Lorenz, 1965) plans and solutions to problems being derived by natural selection, a trial-and-error process acting over evolutionary time (e.g., see Catania, 2010). A Darwinian Creature’s solutions to problems may be “clever,” but this is not the same as attributing to the individual Darwinian Creature the cleverness involved in deriving these solutions to problems. The situation is different with the Skinnerian Creature because, by trial-and-error learning of the relationship between responses and consequences in its own lifetime, the individual Skinnerian Creature derives its own individual solutions to problems (Domjan, 2010).

Popperian Creatures are distinctly different because, instead of solving problems by physically acting in the environment in real time, they derive solutions to problems ahead of time by formulating plans and then by acting on them (Dennett, 1996). As Geffner (2013, p. 341) put it, the Popperian animal is “thinking before acting.” Gregorian Creatures go beyond this by making use of mind tools for solving problems, with this being most prominently seen with human verbal language (Dennett, 1995, 1996).

When we consider Skinnerian and Popperian Creatures as falling on continuums, we can indicate where intelligence and cognition become prominent. When looking for evidence of “intelligence,” the relevant continuum pertains to an individual’s proficiency at flexible problem solving and, following Grush (1997), we envisage “Popperian” as having crossed a threshold into the realm of genuine cognition because these are animals that rely on representations when deriving solutions to specific problems.

At the most basic level, a “representation” can be thought of as something that stands for something else (Webb, 2012) or, more accurately, something that is used to stand in for something else (Grush, 1997). Gallistel (1990a) and later Gallistel and King (2009) emphasized a functional equivalence between internal representations and relevant entities or events in the outside world, with representations serving as theoretical constructs that have a role in cognitive science analogous to the way homomorphism works in mathematics. The emphasis on representation as being critical to cognition is important because this is a step toward understanding how a Popperian Creature can interface with the outside world in a way that goes beyond stimulus-and-response. This allows for foresight, predicting outcomes of plans and acting in ways that flexibly anticipate what is likely to be beneficial rather than relying more strictly on stamped in solutions to problems.

This perspective might make it easier to break free from an intuition that there must be a tight relationship between brain size and intelligence. Where invertebrates fit on a continuum of intelligence is an empirical question and, as Chittka and Niven (2009) illustrated with examples from social insects, often the answer may be considerably different from what is expected. Bees and ants defy the common sense notion that being a mammal or a bird with a large brain is a prerequisite for a substantial level of intelligence. For spiders, we find comparable defiance of common sense among the species in the salticid genus *Portia*.

## The Salticid Brain

As body size gets smaller, it is inevitable that the maximum number of neurons that can be housed in a brain will also get smaller because there is a limit on how small neurons can be and still remain functional (Faisal et al., 2005; Niven and Farris, 2012; Niven and Chittka, 2016). Of course, nobody has ever literally counted the number of neurons in a salticid’s brain, but the estimated number even for much larger spiders is in only the tens of thousands (Babu, 1975; Babu and Barth, 1984). Saying “only” is relevant when we compare this to the brains of large vertebrates. For example, elephant brains are estimated to have 100,000,000,000 neurons and human brains 85,000,000,000 (Eberhard and Wcislo, 2011), but the possibility of spider-sized brains having such a large number is ruled out due to limitations on the extent to which neurons can be miniaturized. This also leads to vast differences in the possible numbers of dendritic connections between neurons. In the human brain, for example, individual neurons often have thousands or tens of thousands of dendritic connections to other neurons (Edelman, 1998), with these being numbers that rival the total number of neurons in a spider’s entire brain.

Interesting possibilities arise when brains are large and, although their focus was not specifically on animal intelligence, Eberhard and Wcislo (2011) suggested that qualitatively different brain processes might be found depending on whether the brain is that of a spider or a primate. In primates, for example, brain functioning can be based on recurrent pathways involving huge populations of neurons and their dendritic connections on a scale that has no parallel in spider-sized brains (Eberhard and Wcislo, 2011). It seems inevitable that, for spider-sized brains, the level at which intelligence-related processes take place will be more at a neuron-to-neuron level instead of at the level of recurrent pathways in large populations of neurons.

Understanding precisely how this and other size-related consequences might influence the expression of animal intelligence seems particularly important when discussing Macphail’s null hypothesis. This hypothesis challenges us to find distinctive instances of different animals using qualitatively different intelligence-related processes. It is in this context that research on *Portia* may become especially relevant.

## The Salticid Spider *Portia*

Found in Africa, Asia, and Australia, 17 species from this genus currently have names and formal taxonomic description (Platnick, 2020). Most of what we know from using *Portia* in research pertaining to intelligence has come from five of these: *Portia africana* and *Portia schultzi* from East Africa, *Portia labiata* and *Portia occidentalis* from Asia, and *Portia fimbriata* from Australia. There are over 6,000 salticid species (Maddison, 2015), with little known about the behavior of most of them, but it seems likely that most salticid species prey primarily on insects, which they capture without the assistance of a web (Jackson and Pollard, 1996). *Portia* cannot be characterized so simply because, besides capturing prey away from webs, *Portia* also builds prey-capture webs and also invades the webs of other spiders where it uses many different prey-specific prey-capture tactics (Harland and Jackson, 2004). The tactics used while in other spiders’ webs include *Portia* using its appendages to move and tense web silk, thereby making signals with which to control the resident spiders’ behavior (Jackson and Cross, 2013).

In and out of webs, *Portia* has an active preference for spiders instead of insects as prey. Besides being potential prey, another spider is, for *Portia*, a potential predator and the risk of the hunter becoming the hunted may have favored reliance on especially flexible prey-capture methods that can be finely tuned to the particular spider being pursued (Jackson and Cross, 2013). Flexibility and fine tuning includes more than *Portia* making web signals and, of particular interest here, it extends to making strategic prey-capture plans ahead of time (Jackson and Cross, 2011).

Something else needs to be emphasized. Learning is typically emphasized when animal intelligence is discussed, often almost as though, by definition, intelligence and learning have to go together (e.g., see Burkart et al., 2017). Yet very little of the research on *Portia* pertains specifically to practice and having prior experience with the problems to be solved. Experiments have repeatedly demonstrated that *Portia* expresses the behavior we envisage as being intelligent without needing to rely on prior personal experience with particular prey or with particular environmental situations for acquiring the information critical to solving problems. On this basis, we conclude that *Portia*’s behavior in experiments is innate (see O’Neill, 2015), but being innate does not mean inflexible or non-intelligent. This is something we will illustrate by reviewing research based on using three particular experimental approaches in which training and learning are not part of the procedure.

## Pre-Planned Detours

Part of *Portia*’s strategy when preying on other spiders is often to adopt an indirect path (i.e., a detour) leading to an optimal location from which to launch an attack (Jackson and Wilcox, 1993), and findings from laboratory experiments imply that *Portia* can make strategic detouring decisions ahead of time. This includes decisions related to the risk of being attacked by the prey spider (Jackson et al., 2002), decisions related to whether a more direct path is available (Cross and Jackson, 2019) and choosing between two indirect paths, with only one leading to prey (Tarsitano and Jackson, 1997; Cross and Jackson, 2016). In each of these studies, the apparatus and the testing protocol were designed with respect to a specific objective of looking for evidence of planning.

**Figure 1:**
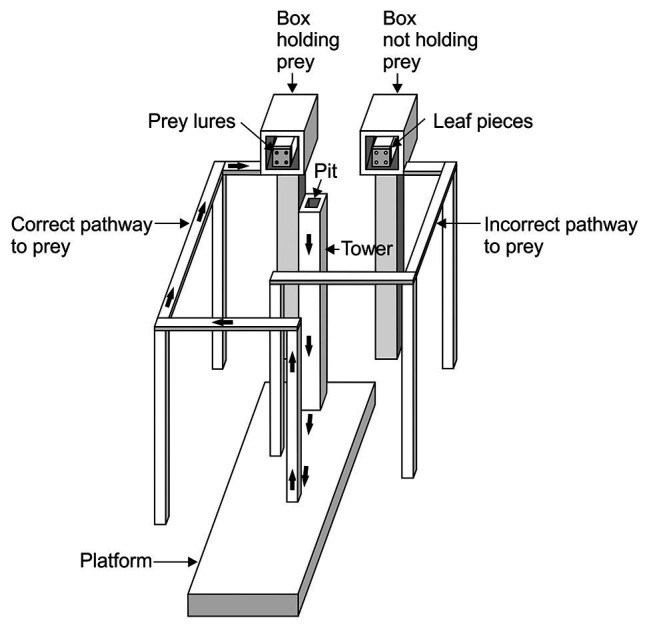
Example of apparatus used in detour-choice experiments. Trial began with a test spider walking out of the pit and on to top of the tower where it could view two boxes. One box contained four lures made from *Oecobius amboseli* and other box contained lures made from four green-leaf pieces. Which box contained prey was determined at random. After the test spider left the pit and walked down from the top of the tower, all of the lures were removed from the apparatus. To complete a successful trial, the test spider chose a walkway after it left the tower and then walked across the platform. The thick arrows indicate the path the test spider took from the tower to the beginning of the correct walkway and then to the end of that walkway. The apparatus sat in a shallow pan filled with water (not shown). Drawing modified from Cross and Jackson (2016).

We will focus on Cross and Jackson (2016) here. In this study (Figure 1), each trial began with *Portia* (the “test spider”) on the top of a tower from which it could view two displays and two pathways, with one pathway leading to a display where there were lures made from prey spiders and the other pathway leading to a control display (dead leaves that were similar in size to the lures). The displays were out of reach from the tower. Moreover, the tower and pathways were on a platform which, in turn, sat in a shallow pan of water, and *Portia* is averse to getting wet. This is important because it meant that the only way *Portia* could reach the lure display without getting wet was to first walk down from the top of the tower to the platform, walk directly away from the location of the lures to arrive at the pole where the correct pathway began (i.e., the pathway that led to the lures) and then continue along this pathway to the display. *Portia* needed to plan ahead because, once it left the tower, the lures and control leaves were removed from the displays, meaning that the test spider could no longer navigate on the basis of seeing the location of the lures. Yet, in this study (Cross and Jackson, 2016), 251 test spiders chose the correct pathway and only 15 test spiders chose the incorrect pathway.

Training and learning were not part of this experimental design; each test spider was used in a single trial and test spiders had no prior experience with the apparatus or testing protocol, so they could not use trial and error. It is a common fallacy to assume that “innate” must imply “inflexible.” However, these experiments, having been specifically designed as ways to look for innate capacity to plan detours ahead of time in a single trial, are a striking illustration of a capacity related to intelligence that is highly flexible and also innate.

*Octopus*, turtles, dogs, and cats – animals much larger than, and only distantly related to, *Portia* – have also been the test subjects in detouring experiments (Kabadayi et al., 2018), but the objectives and methods were substantially different. Typically, these were experiments in which a test subject viewed a target of interest (e.g., food) behind a see-through barrier (e.g., a glass sheet or a wire fence) and the target could not be directly accessed (e.g., Smith and Litchfield, 2010). The test subject’s typical response was to make repeated unsuccessful attempts to access this target directly and, when the test subject finally succeeded, it is only by moving around the barrier. It may be easy to envisage this as the test subject having a “eureka moment” in which it suddenly accepted that its efforts to go directly to the target were futile and that, in this eureka moment, it recognized a detour was a workable alternative (Jones, 2003; Chronicle et al., 2004).

This is almost the exact opposite to the way *Portia* behaved (Cross and Jackson, 2016), and these experiments were designed very differently. For instance, repeated efforts to leap directly toward the lures was absent from these experiments. There was nothing suggestive of a eureka moment and, instead, a more accurate characterization may be that *Portia* first assessed the situation and then acted on a plan from the beginning, with this being a spontaneous plan requiring no prior training with the experimental apparatus or protocol. The detouring experiments reviewed by Kabadayi et al. (2018) appear to be especially good for finding evidence of impulse control, but there was little to suggest *Portia* having an impulse-control problem to solve. Impulse control seems to be more aligned with operating as a Skinnerian Creature, but the *Portia* detouring experiments were designed instead as a way of looking for evidence of a kind of intelligence that Popperian Creatures express. These are the Creatures that spontaneously find solutions to problems by internal processing instead of having to first try out potential solutions by actually acting in the physical environment.

## Expectancy Violation

Macphail (1985) argued that comparisons should be made between animals that occupy contrasting ecological niches because of how different animals adapt to the specific demands of the particular environments in which they live. We can consider this argument in the design of expectancy violation experiments, in which pre-verbal infants (e.g., Wynn, 1992), non-human primates (e.g., Hauser et al., 1996) and even parrots (e.g., Pepperberg and Kozak, 1986) have been the typical subjects. However, Pepperberg (2002) argued that, as long as the methodological details are tailored to the biology of the particular species being investigated, expectancy-violation methods should be applicable to a wide taxonomic range of animals. To date, very little has been done to investigate expectancy violation by an arthropod but, consistent with Pepperberg’s argument, *Portia*-specific expectancy violation methods were used successfully in research on *P. africana*.

In expectancy-violation experiments, it is customary to let a test subject preview a scene that disappears and then, at a later time, comes into view again (Shettleworth, 2010). For example, a screen might be put between the scene and the test subject and then, during the time when the test subject’s view is blocked, a scientist can alter the items in the scene. Data relevant to expectancy violation come from comparing how test subjects respond to altered scenes with how they respond to scenes that stay the same. Instances of subjects gazing at an altered scene for longer than they gaze at an unaltered scene (i.e., instances of longer “looking time”: see Winters et al., 2015) have been typically regarded as evidence that the subject has detected a mismatch between the current scene and a representation of a scene it had previously loaded into working memory (i.e., this has been a basis for concluding that the individual has experienced expectancy violation).

The expectancy-violation experiments using *P. africana* have been designed to take advantage of how these spiders have exceptional eyesight for animals of their size (Harland et al., 2012), as well as how they respond to lures in similar ways to how they respond to living prey (Jackson and Cross, 2011). These experiments also took advantage of how, in their natural habitat, these spiders pay attention to the different features of their prey (Harland et al., 2012), they routinely take detours to reach prey (Tarsitano and Jackson, 1997; Cross and Jackson, 2016), and they encounter various numbers of other conspecific individuals (Nelson and Jackson, 2012). However, the data of interest when using *P. africana* differ from the standard “looking time” used in experiments on bigger animals.

**Figure 2:**
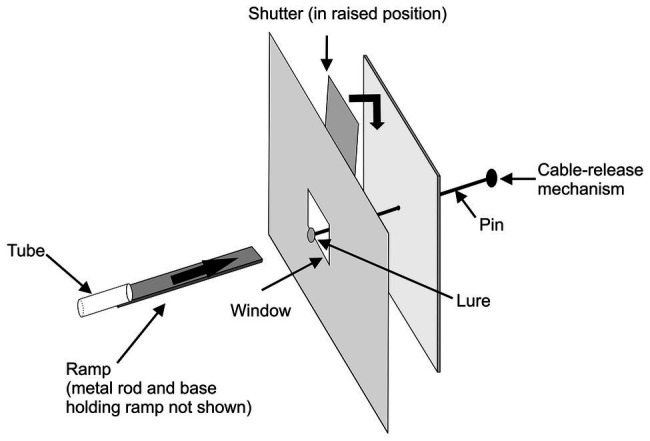
Apparatus used in expectancy-violation experiments for changes in prey type. A trial began with a test spider leaving the glass tube and walking across the ramp (thick arrow) toward a lure. Once the spider had faced the lure for 30 s, the lure was pulled back from the window and the shutter was lowered for 90 s, blocking the spider’s view of the lure. The lure was removed from the pin during the 90 s. In experimental trials, a different lure was then attached to the pin and, in control trials, the same lure was re-attached to the pin. After the 90 s, the shutter was raised, and it was recorded whether the test spider leapt at the lure. Reprinted by permission from Springer (Cross and Jackson, 2014).

**Figure 3:**
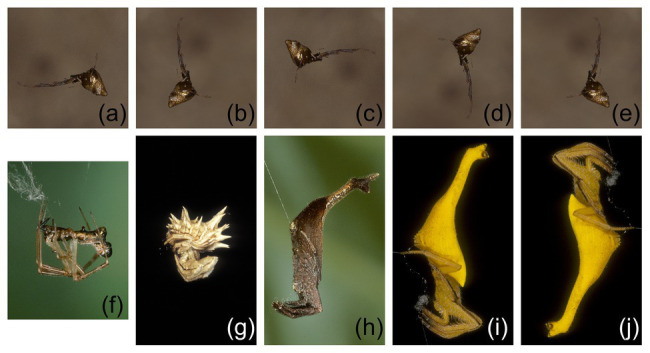
Prey spiders used for making lures in expectancy-violation experiments for changes in prey type. **(A–E)** *Argyrodes* sp. 1 positioned in different orientations; **(F)** *Argyrodes* sp. 2; **(G)** *Pycnacantha tribulis*; **(H)** *Arachnura scorpionoides* (brown morph); **(I,J)** *Arachnura scorpionoides* (yellow morph) positioned in different orientations. Reprinted by permission from Springer (Cross and Jackson, 2014).

In the first study (Cross and Jackson, 2014), experiments were designed to determine whether *P. africana* holds representations of specific prey types in working memory during predatory sequences (Figure 2). After seeing a particular prey item at the beginning of a trial (Figure 3), *Portia* positioned itself for initiating an attack, but then, before *Portia* could act, the prey item was hidden behind a shutter for 90 s. During this time, *Portia* waited while facing the shutter and then, once the shutter was lifted after this delay, *Portia* could see prey that was either identical to or different from the type of prey it had seen earlier.

**Figure 4:**
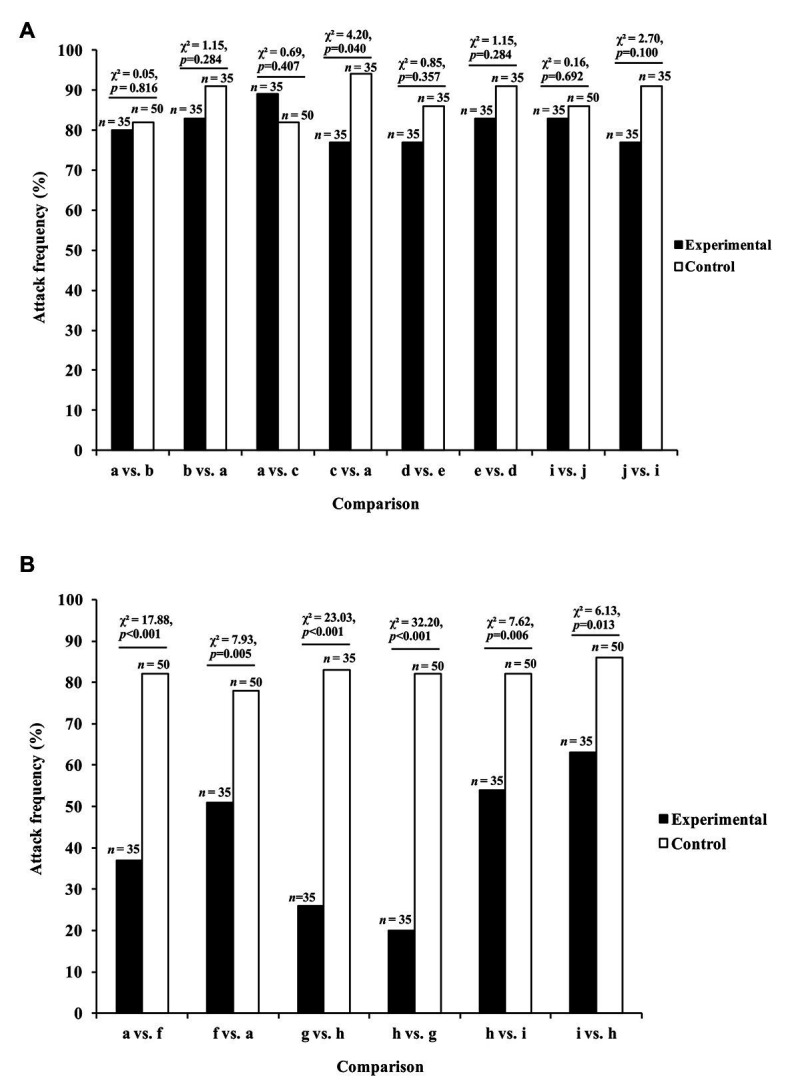
Results from expectancy violation experiments in which *Portia africana* was tested with **(A)** different prey orientations (prey type remained constant) and **(B)** different prey types (prey orientation remained constant). See Figure 3 for the different prey orientations and types shown during trials. Experimental trials: first prey orientation or prey type replaced by second prey orientation or prey type. Control trials: first prey orientation or prey type did not change during trial. Data analyzed using *χ* ^2^ tests of independence. Attack frequency: percentage of test spiders that leapt at the prey. Total number of test spiders (*n*) shown above bars. Reprinted by permission from Springer (Cross and Jackson, 2014).

In these experiments (Cross and Jackson, 2014), the data of interest were the number of test spiders (*P. africana*) that attempted to attack this lure, instead of looking time (i.e., instead of the length of time the test spider spent gazing at the lure). There was no evidence that *Portia* was more or less likely to attack if only a lure’s orientation had changed during a trial (Figures 3A–E,I,J, 4A). However, when the prey species (Figures 3A,F–I) or prey color (Figures 3H,I) had changed during the trial, significantly fewer *Portia* individuals followed through with an attack (Figure 4B). All of these experiments were counterbalanced, and there was no evidence to suggest *Portia*’s responses were influenced by the order in which prey were presented. This suggests that *Portia* experienced expectancy violation when the representation of the prey type it had loaded into working memory at the beginning of a trial did not match with the prey it saw later.

**Figure 5:**
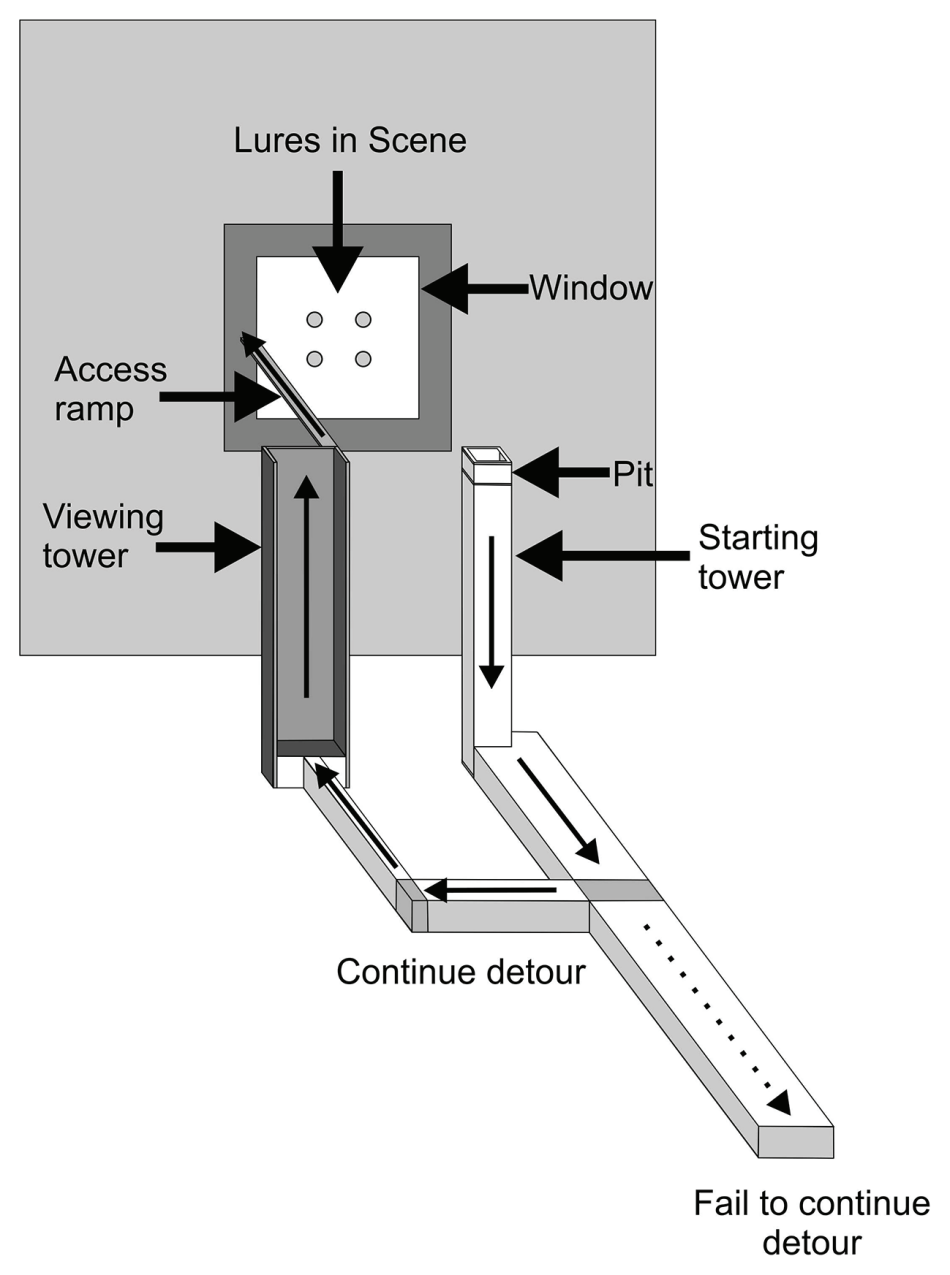
Apparatus used in expectancy-violation experiments for changes in prey number (apparatus sat in a shallow pan filled with water; not shown). A trial began with a test spider walking out of the pit and on top of the starting tower. The thin arrows indicate the path that test spiders then took to reach the lures without getting wet; the dotted arrow indicates the path that test spiders took to opt out of completing the detour. When the test spider arrived at the bottom of the viewing tower, which was opaque, the scene was removed and then replaced by a different scene or else the previous scene was returned. It was then recorded whether the test spider crossed the access ramp after reaching the top of the viewing tower. Drawing modified from Cross and Jackson (2017).

In the second study (Cross and Jackson, 2017), experiments were designed to determine whether *P. africana* represents the specific number of prey in a scene (Figure 5), with the methods required for this being substantially different from the methods in the earlier study (Cross and Jackson, 2014). In these experiments, *Portia* had to complete a detouring task, and the data of interest pertained to whether *Portia* became less inclined to complete the detour when presented with an unexpected number of prey at the end of the detour. These experiments began with *Portia* leaving a pit and standing on top of a starting tower from which it could view a scene consisting of a particular number of prey items. In order to reach this scene, *Portia* walked down from the starting tower before it walked across a walkway, up a viewing tower and then across an access ramp. However, when walking up the viewing tower near the end of the detour, the scene went out of *Portia*’s view because the walls of this tower were opaque. The scene was either changed or it remained the same during the time that *Portia* walked up the viewing tower. It was only after reaching the top of the viewing tower when *Portia* could view the scene again, but now the number of prey items might have changed.

**Figure 6:**
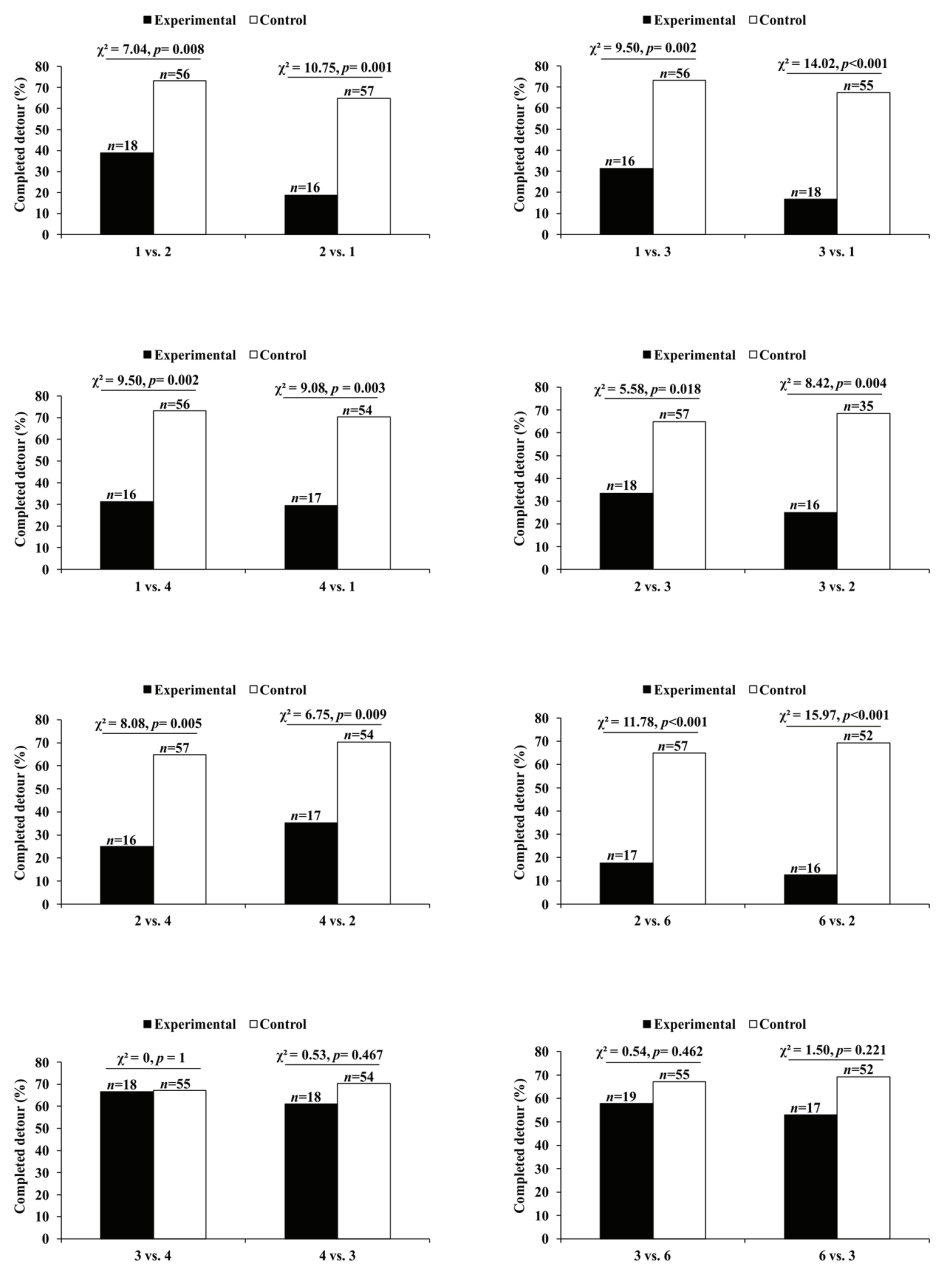
Results from expectancy violation experiments in which *P. africana* was tested with different numbers of prey. Experimental trials: first prey number replaced by second prey number. Control trials: first prey number did not change during trial. Data analyzed using *χ* ^2^ tests of independence. Completed detour: percentage of test spiders that crossed the Access Ramp to reach the location of the prey. Total number of test spiders (*n*) shown above bars. Figure modified from Cross and Jackson (2017).

Compared with control trials in which the number of prey seen was the same as before, *Portia* became disinclined to complete the detour when the following changes in number were made: 1 vs. 2, 1 vs. 3, 1 vs. 4, 2 vs. 3, 2 vs. 4, or 2 vs. 6 (Figure 6). These effects were independent of whether the larger number of prey was seen at the start or at the end of the trial. Moreover, when the number remained the same during a trial, there was no evidence that changing the size or arrangement of the prey influenced *Portia*’s inclination to complete the detour (see Cross and Jackson, 2017). There were also no significant effects when the number of prey changed between 3 vs. 4 and 3 vs. 6 (Figure 6), which suggests that *Portia* may characterize three or more prey as a single category (“many prey”). However, *Portia* seems to represent 1 and 2 as discrete number categories.

The range over which *Portia* represents prey number appears to be consistent with the range over which vertebrates have been shown to practice subitizing, this being the rapid and accurate estimation of small numbers of individuated objects (Davis and Pérusse, 1988). Yet the way *Portia* responded in experiments is inconsistent with how subitizing is usually characterized. For instance, primates (Hauser et al., 1996) normally respond no more than a few seconds after viewing a stimulus, but *Portia* normally responded after a minute or longer. *Portia* typically engages in a slow, methodical visual-inspection routine before responding (Harland et al., 2012), which is also inconsistent with how subitizing is normally characterized as being automatic and pre-attentive. We propose that, instead of subitizing, *Portia* slowly individuated objects and then held separate representations of these objects in working memory. More specifically, we propose that, while on top of the first tower, *Portia* loaded a representation of the number of prey individuals in the scene into working memory and then, while on top of the second tower up to 21 min later, *Portia* compared the number of prey in the scene now in view with the number of prey represented while on the first tower.

## Solving a Novel Confinement Problem

When discussing Macphail’s null hypothesis of no differences in intelligence, we need to specify the kind of difference being considered. Much of the time, it seems implicit that the issue of interest is the level to which intelligence is expressed in a domain-general manner. However, the extent to which cognitive processes used by animals are domain-specific instead of domain-general remains poorly understood (Chiappe and MacDonald, 2005; Anselme, 2012), and arguments that improving capacity for domain-general intelligence requires a higher investment in mass of expensive brain tissue (Burkart et al., 2017) suggests that especially small animals, including spiders, will be skewed more toward domain-specific intelligence than is the case for larger animals such as birds and mammals (Logan et al., 2018). Research on *Portia* may be especially interesting in this context.

Part of *Portia*’s strategy for targeting web-inhabiting spiders as prey involves using signals to gain dynamic fine control over the resident spider’s behavior (“aggressive mimicry”; Jackson and Cross, 2013). This is achieved by *Portia* using any one or a combination of its 10 appendages (eight legs and two palps) to generate web signals (i.e., vibration and tension patterns on the silk lines in the web). Sometimes *Portia*’s signals may be readily understood as mimicking the movements of a small insect trapped on the web; in these instances, *Portia* lures its victim over for the kill. The variety of signals at *Portia*’s disposal seems unlimited; the way any one appendage moves can vary and *Portia* can move each appendage independent of how other appendages are moving (Jackson and Blest, 1982). By repeating signals that elicit an appropriate response from its intended prey and by trying new signals when an appropriate response is not forthcoming (Jackson and Wilcox, 1993; Jackson and Nelson, 2011), *Portia* achieves a high level of proficiency at adjusting its predatory strategy to the particular prey spiders it encounters. Using this trial-and-error strategy (a “generate-and-test algorithm”; Simon, 1969), *Portia* preys on a vast array of different kinds of spiders (Jackson and Pollard, 1996), including spiders that can prey on *Portia*. It has been proposed that *Portia*’s capacity for flexibly deriving signals by trial-and-error is an important adaptation for successfully targeting prey that are also predators (Jackson, 1992).

**Figure 7:**
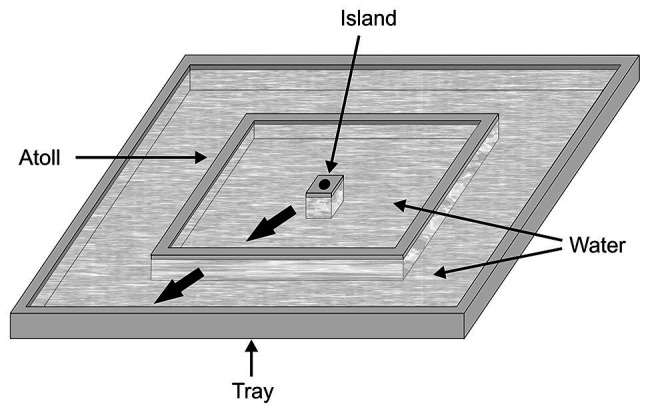
Apparatus used for ascertaining whether spiders can solve a novel confinement problem by using trial-and-error. In a water-filled tray, there was an island surrounded by an atoll. The test spider emerged from a hole in the island and then either chose to leap or swim away from the island. Before testing began, it was determined at random which of these two potential choices would succeed. When the test spider made the successful choice, it was helped across to the atoll, and a record was made of whether the test spider repeated its choice to reach the edge of the tray (thick arrows). When the test spider made the unsuccessful choice, it was forced back to the island, and a record was made of whether the test spider switched its choice when attempting to reach the atoll again. Reprinted by permission from Springer (Cross and Jackson, 2015).

Whether *Portia*’s proficiency at using trial-and-error is restricted to this predatory strategy (domain specific) or whether it is applicable to novel problems (see Beecher, 1988) has been considered in experiments where individuals were faced with a problem of how to escape from an island in a water-filled tray (i.e., a confinement problem; Figure 7). The island, in the middle of the tray, was surrounded by an atoll; water filled the space between the island and the atoll, and also filled the space between the atoll and the edge of the tray. The basis for calling this problem “novel” includes how there is no evidence that *Portia* routinely crosses water in nature. Moreover, adding to the novelty of the problem, test spiders were helped forward across the water to the atoll or forced back to the island during the experimental trials. There were only two ways *Portia* could leave the island, either by stepping into the water and then swimming the whole way across to the atoll or by first leaping into the water and then by swimming. When leaving the island by swimming, test spiders slowly placed their forelegs on the water, pushed off from the island with their rear legs, moved completely out into the water in a spread-eagle posture and then propelled their bodies across the water surface by moving their legs in a stepwise fashion (see Suter, 2013). When leaving the island by leaping, spiders landed on the water at a point about halfway across, and then swam the rest of the way to the atoll.

*Portia* individuals were assigned at random to two groups, with these two groups differing with respect to the method of leaving the island that would be successful. When *Portia* used the escape method pre-determined for its group to succeed, a small plastic scoop was used to make waves behind *Portia* to help it across to the surrounding atoll. When *Portia* used the other escape method, the scoop was used to make waves to move *Portia* back to where it had started from. Once on the atoll or back on the island, test spiders could then try again. In the first experiments based on this confinement problem, the test spiders were *P. fimbriata* (Jackson et al., 2001). The test spiders that had succeeded at arriving on the atoll usually repeated the same escape method to then reach the edge of the tray and those that failed usually switched to using the other escape method.

**Figure 8:**
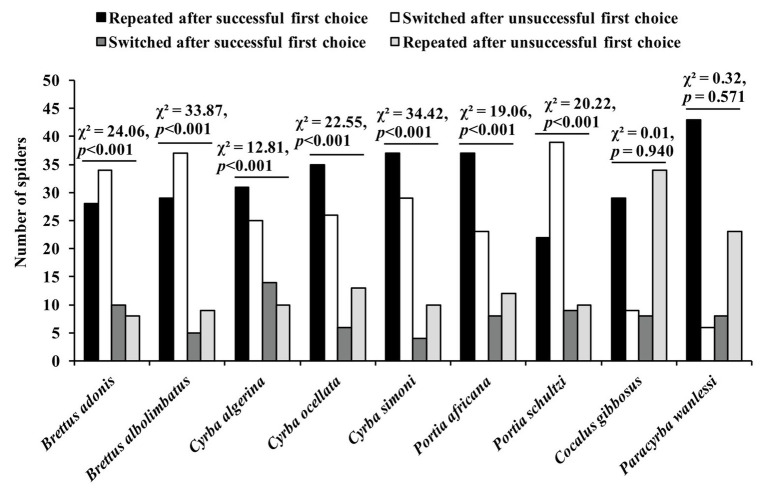
Results from confinement experiments. Spider began trial on an island surrounded by an atoll in a pan of water and given two opportunities to choose its method of crossing the water (i.e., by leaping or by swimming). Successful first choice: plastic scoop made waves to help spider across to the atoll. Unsuccessful first choice: plastic scoop made waves to move spider back to the island. After making its first choice, it was recorded whether the spider repeated that choice or switched. Data analyzed using *χ* ^2^ tests of independence. Reprinted by permission from Springer (Cross and Jackson, 2015).

In a more recent study using the confinement problem (Cross and Jackson, 2015), two other *Portia* species (*P. africana* and *P. schultzi*) were used as test spiders, and there were also seven other test-spider species from different genera, but with all of these genera being from the same salticid subfamily (*Spartaeinae*) as *Portia*. Five of the non-*Portia* species (*Brettus adonis*, *Brettus albolimbatus*, *Cyrba algerina*, *Cyrba ocellata*, and *Cyrba simoni*), along with the two *Portia* species, are known to practice aggressive mimicry and derive signals by trial and error, whereas the other two non-*Portia* species (*Cocalus gibbosus* and *Paracyrba wanlessi*) are not known to practice aggressive mimicry. The findings from experiments showed that the seven aggressive-mimic species were proficient at solving the novel confinement problem by repeating “correct” choices (i.e., the choices that delivered them to the atoll) and by switching when they made “incorrect” choices (i.e., the choices that sent them back to the island), but there was no evidence of the two non-aggressive-mimic species solving the same novel problem (Figure 8). These findings suggest that species which use trial and error to solve aggressive mimicry problems are predisposed to be proficient at using trial and error in a novel context.

Local adaptation is another relevant factor because different populations of a single *Portia* species are known to adopt different predatory strategies (Jackson and Pollard, 1996). For example, two populations of *P. occidentalis* from the Philippines have been investigated (Los Baños and Sagada). Los Baños is a low-elevation rainforest habitat where the range of prey-spider species is much wider than in Sagada, a high-elevation pine-forest habitat, and it was found that individuals from Los Baños were significantly more inclined than individuals from Sagada to derive web signals by trial-and-error (Jackson and Carter, 2001). In the context of domain-generality, there was another interesting difference. Individuals from Los Baños were significantly more inclined than individuals from Sagada to solve the novel confinement problem by trial-and-error (Jackson et al., 2006).

These findings from different species, and from different populations of a single species, appear to be salticid examples of a transition from domain-specific to domain-general problem-solving capacity, this being a transition also suggested as happening sometimes with other animals (Johnston, 1982; Papaj, 1986; Dukas, 1998), but we should be wary of envisaging domain-specific and domain-general as two distinct categories. A more useful alternative is to envisage “domain specific” and “domain general” as being terms pertaining to different ends of a continuum (e.g., see Jackson and Cross, 2011). The findings from the confinement problem experiments suggest that the domain-general region of this continuum is particularly relevant for understanding the behavior of aggressive-mimic spartaeines. In other words, being proficient at solving a novel confinement problem by trial-and-error may be a spin-off from these spiders having evolved proficiency at deploying highly plastic aggressive-mimicry strategies in the context of predation.

## Are Spiders Intelligent?

When discussing his null hypothesis, Macphail (1985, 1987; Macphail and Bolhuis, 2001) focused almost entirely on vertebrates and he had little to say about arthropods (insects, spiders, crustaceans and their kin). This drastically limited the scope of his hypothesis since only a small minority of animal species are vertebrates; most animal species are arthropods. Yet, while this omission is jarring to some of us (e.g., see Kupfermann, 1987), it may be hardly noticed or else assumed to be obviously justified by others.

To decide how serious this omission might be, it may help first to ask how the null hypothesis might actually be useful. How we answer this question is similar to how Zentall (2018) answered a comparable question about Morgan’s canon. Rather than being a call to reduce intelligence to its lowest common denominator, the null hypothesis will be more useful when seen as a way to challenge investigators to develop procedures for identifying differences in intelligence. When we refer to “intelligence,” the core topic of interest is flexible problem-solving capacity, and especially the distinction on a continuum between domain generality and domain specificity. The null hypothesis can then be useful in challenging us to develop procedures for objectively specifying levels of flexibility and domain-generality.

This may work as a rationale for the null hypothesis, but not with arthropods relegated to a footnote. Even if not explicitly stated, a decision not to include arthropods in the conversation often seems to be based on accepting as a foregone conclusion that, at best, arthropod intelligence is distinctly inferior to vertebrate intelligence or, at worst, arthropods are not intelligent at all (i.e., arthropods are automatons).

For examples of arthropod intelligence, we have focused especially on *Portia*, but we should point out that cognition, instead of intelligence, was the context in which the research we reviewed was originally discussed. This is important because we envisage cognition, which pertains to representation, and intelligence, which pertains to flexibility, as being overlapping, but not identical, topics. *Portia* gets our attention because we are especially interested in instances in which well-developed cognitive capacities are deployed in flexible problem solving.

Flexible problem solving is not necessarily cognitive in any substantial way. To suggest otherwise would be to forget radical behaviorism’s explicitly non-cognitive interpretation of operant conditioning. This behaviorist interpretation may have faded with time but, at the very least, it shows that non-cognitive intelligence, or flexible problem solving, by animals is conceivable. In Dennett’s scheme, these animals are Skinnerian Creatures. When we consider Darwinian Creatures, which might be aptly called automatons, non-cognitive intelligence is also relevant, but in a different way. These animals use solutions derived by natural selection, a non-cognitive flexible problem-solving algorithm with formal similarities to operant conditioning (Skinner, 1981; Watson and Szathmáry, 2016). It is with Popperian Creatures that the expression of flexible problem solving becomes distinctively cognitive in character.

When defining intelligence, Burkart et al. (2017) emphasized individuals showing proficiency at acquiring new knowledge from interacting with the physical or social environment. This might seem more characteristic of a Skinnerian Creature rather than a Popperian Creature, but the extent to which Burkart et al. (2017) allude to knowledge, understanding and representation would probably go well beyond anything a radical Skinnerian would accept. Burkart et al. (2017, p. 2) also refer to using “this knowledge to organize effective behavior in both familiar and novel contexts” and, by saying this, they imply that learning-based intelligence is cognitive in character, this being aligned with post-Skinnerian representation-based theory of learning (see Gallistel and King, 2009) and, as such, more related to Popperian than Skinnerian Creatures.

Intelligence and learning are often discussed together, but making learning a necessary part of the definition of “intelligence” would artificially remove the research we reviewed on *Portia* from the conversation. For this research, each individual test spider was a subject in a single trial and all of the test spiders had been reared under standardized conditions in the laboratory with no prior experience of the procedures and apparatus. The rationale for these procedures was to ensure that test spiders were not solving the experimental problems as Skinnerian Creatures. In the detour-choice experiments, for example, the test spider solved the problem by choosing a particular path without having had any prior experience of the consequences of taking that path, which is not compatible with being a Skinnerian Creature. The findings from the detour-choice experiments are also incompatible with test spiders being Darwinian Creatures because the particular path that spiders took to solve the detouring problem was set at random before each trial began.

It might be disconcerting that we say “innate” because this word is often associated with the idea of animal being an inflexible automaton, as though being innate is the antithesis of being intelligent or cognitive. To understand why this is not the case, there is an important distinction to make between having solutions to problems and having the capacity to find these solutions. When presented with detour-choice problems, for instance, *Portia* uses an innate and flexible problem-solving capacity. In other words, the capacity to solve detour-choice problems is innate, but the specific solutions to these detour-choice problems are not innate and they are also not memorized solutions derived from prior personal experience.

The setting in which *Portia* encounters prey is typically accompanied by extreme unpredictability and mortal risk, and this may be a major component of the adaptive context in which these flexible capacities evolved. When invading other spiders’ webs, *Portia* enters the prey-capture arena of another predator and, when making web signals, intimately interfaces with that predator’s sensory system. From *Portia*’s perspective, encounters that end with *Portia* killing the resident spider are successful and encounters that end with the resident spider killing *Portia* are unsuccessful. Success often depends on *Portia* gaining dynamic control of the resident spider’s behavior by deploying especially intricate and flexible behavior that is cognitive and intelligent in character (Harland and Jackson, 2004; Jackson and Cross, 2011, 2013). Burkart et al. (2017) emphasized the role of social unpredictability in the evolution of general intelligence, which is interesting because social unpredictability occurs when groups of conspecific individuals are actively engaged in complex interactions. This seems similar to the unpredictability *Portia* contends with while engaged in intricate and intimate interactions with other predators.

Burkart et al. (2017) also envisaged general intelligence as being closely related to three core executive functions: working memory (see Baddeley, 2012), cognitive flexibility, and inhibitory control, with inhibitory control including selective attention as well as behavioral inhibition and cognitive inhibition. Interest in all of these executive functions has been integral to research on *Portia* (Jackson et al., 2002), as well as other salticids (Jackson and Cross, 2011). Research on *Evarcha culicivora* (Cross and Jackson, 2009, 2010a,b) has been especially relevant. This salticid specializes at preying on mosquitoes and, for this salticid, specialization includes intricate, innate systems of deploying selective attention to specific types of prey. This includes specific odors priming selective olfactory attention and specific optical cues priming selective visual attention, and also cross-modality priming in both directions (selective visual attention being primed by specific odors and selective olfactory attention being primed by specific visual stimuli).

To more fully address whether salticid species differ with respect to domain generality, it is important to determine whether there are correlations in how individuals perform in certain tasks. It would be interesting to investigate, for instance, whether superior proficiency at deploying selective attention is correlated with superior performance on other tasks, including solving novel problems and planning detours. Computational complexity may also be especially relevant when characterizing animal intelligence and potentially measuring how intelligence varies among species and populations within species. This may be especially tractable when focusing on converging topics, such as expectancy violation with respect to a change in number.

## Representation of Number

Numbers are related to mathematics and, being abstract, mathematics is often experienced by people as a hard subject. Perhaps this is why we tend to admire people who are good at mathematics and why even a hint of mathematical aptitude by a spider can seem sensational. However, relying on impressions alone will not take us very far toward a goal of understanding number-related capacities in the context of animal intelligence. Conceptual clarity is especially important whenever intelligence and cognition are discussed, but it is also especially difficult when discussing numerical cognition in particular. Returning to *Portia*’s performance in the expectancy-violation experiments, we can begin by focusing on two of the most basic questions that arise when we propose that this is an example of cognition-based intelligence. If this is cognitive, then what is represented? If the answer to that question is something related to numbers, then what kind of number do we mean?

In these experiments (Cross and Jackson, 2017), test spiders could see prey at the beginning and at the end of a detour path, but not while taking the detour. The number of prey in view at the end was either the same as or different from the number in view at the beginning, and the primary finding was that test spiders hesitated at the end of a detour when the number was different. This basic finding held even when there were control trials for considering the possibility of non-numerical variables related to prey size and prey arrangement being alternative explanations.

Based on the experimental findings, we can offer an answer to the question of what is represented by saying it is the number of prey individuals in a scene. We can also specify the kind of number we mean, but we have to do this carefully because, all too often, we have been forced to guess how number-related expressions are used in the vast scientific literature. The first step is to be mindful of the word “of” when we propose that *Portia* represents the “number of prey.” The prey are individual physical things, but numbers are abstract. The type of number we mean when saying “the number of prey” is important, but whether *Portia* literally represents numbers as strictly abstract constructs is not the specific question the expectancy-violation experiments addressed.

We can begin with the casual expressions “counting numbers” and “measuring numbers” before moving on to kinds of numbers as formally-defined in mathematics. For doing this, we can envisage the expectancy-violation experiments as presenting test spiders with a problem of determining how many prey were seen at the end of a detour and whether this is the same as or different from how many prey were seen before. We say “how many” because the kind of answer we expect is a counting number (1, 2, and so forth), implying discrete countable things. For *Portia*, the countable things were individuated objects or, more specifically, prey individuals, but saying “countable” is not the same as saying “counted.” “How much” implies a different kind of problem, with the answer being expressed using measuring numbers (i.e., the kind of number that applies to a continuum). “How much” pertains to stuff that is measured, not counted. Our hypothesis is that, instead of representing “how much” prey stuff is in a scene, *Portia* individuates prey items and represents “how many” prey individuals are in a scene.

As Gregorian Creatures, we may rarely think about how remarkable it is that we express both “how many” and “how much” using numbers. In mathematics, the abstract analogues of the counting numbers are the positive integers (1, 2, …). All numbers are abstract, but the rationale for the abstracting that leads to the positive integers comes from focusing on the concrete action of individuating objects. Owing to this focus on correspondence, “natural numbers” is an appropriate name for the positive integers (Rucker, 1987).

These are the numbers used for doing basic arithmetic (addition, subtraction, multiplication, and division), and it is with further abstraction that we derive the rational numbers and then the real numbers, which we use for expressing magnitudes on a continuum. The progression leading by abstracting to the real numbers began with the natural numbers during the history of mathematics and this also appears to be the typical progression in the development of numerical comprehension and proficiency by children (Carey and Barner, 2019). As Gregorian Creatures, mathematicians and children do this abstract work using an arsenal of mind tools inclusive of verbal language, numerals, decimal places, equations, formal logic, and so forth (Rucker, 1987).

It is here that we need to be especially careful when discussing non-human animals, including *Portia*. Macphail’s null hypothesis challenges us to look for qualitatively distinct kinds of intelligence, and the abstract, flexible problem-solving capacity supported by mathematics as mind tools seems to be a prime candidate. Animals expressing this kind of intelligence are Gregorian Creatures, but the findings from using *Portia* in expectancy-violation experiments are not evidence of *Portia* engaging in abstract numerical reasoning as a Gregorian Creature.

However, *Portia*’s performance in these experiments is comparable to the performance of pre-verbal human infants in similar experiments (Carey, 2004). For *Portia*, as for a 1-year-old infant, this capacity does not seem to be applied beyond three individuated objects. Yet, as Carey (2004) points out, this is a non-trivial cognitive capacity and it seems to be an innate cognitive precursor to the abstract derivation of integers and then the other numbers. The way the expression “exact” corresponds to integers as abstract constructs is similar to the way “exact” corresponds to individuated objects. Owing to the experimental methods, the findings for *Portia* corresponded with “exact” in this context related to individuating. *Portia* displayed evidence of expectancy violation when the scene in view at the end of a detour, compared with the scene at the beginning of a trial, had one more prey individual and also when it had one fewer prey individuals. However, these experiments using *Portia* seem to differ considerably from much of the literature pertaining to non-human animals displaying number-related capacities.

Conclusions from more familiar research on animals displaying number-related cognitive capacities tend to be based on correspondence to the Weber-Fechner law and referred to as instances of animals using an “approximate number system” (e.g., Nieder, 2019). Although the Weber-Fechner law, and expressions such as “quantity” and “amount,” can be relevant when comparing scenes populated by discontinuous objects, the Weber-Fechner law is not about individuating objects as directly as is the case when *Portia* was investigated using expectancy-violation methods.

The Weber-Fechner law pertains to finding that the discriminability of two magnitude values is a function of their ratio (Nieder, 2019). The magnitudes relevant to this law include, for example, brightness, loudness, duration, length, and area, all of which are normally envisaged as continuous variables. Real numbers, as highly abstract constructs, can be applied to continuous variables, but it is apparent that this is not the kind of number intended when a system used by an animal is called the “approximate number system.” In better designed experiments, the animal compares scenes populated by objects, and considerable effort is made to rule out the influence of continuous variables on experimental findings. This leads to conclusions pertaining to number, but now from a perspective different from expectancy-violation experiments using *Portia* and preverbal infants.

The perspective we have when considering the approximate number system is relevant to intelligence, cognition, and numbers, but with the sense in which it pertains to numbers seeming less direct and less specific. Reference to ratios might suggest that, when applied to scenes populated by objects, the cognitive capacity revealed by correspondence to Weber-Fechner law is a precursor to understanding fractions and the rational numbers expressed to decimal places. Saying “approximate” would seem appropriate because, although all rational numbers are discontinuous, there is no conceptual end to how small they can be, which in turn means rational numbers correspond at least roughly to answering “how much” questions with measuring numbers. Another logical possibility is that, when using the approximate number system, the animal renders a representation corresponding to a specific natural number, but with an accompanying representation of a level of uncertainty. However, trying to answer questions about the intended kind of number might be misguided because the major distinction seems to be between individuating as primary versus ratios as primary, with this distinction being more fundamentally important than is widely acknowledged (Gebuis et al., 2016). It may be only Gregorian Creatures that can achieve the level of abstract reasoning needed to bring about a convergence of the different concepts of number implicit in the distinction between individuating as primary and ratios as primary.

## Spatial Navigation

Spatial navigation may be a more rewarding context in which to investigate the intelligence-related capacities animals display specifically with respect to “measuring numbers.” This could be especially interesting with respect to abstract intelligence because, when based on path integration, spatial navigation implies computationally complex behavior, by which we mean behavior that appears to require the equivalent of mathematical calculation by the animal (Gallistel, 1990b; Grace et al., 2020).

Path integration by arthropods has been investigated especially often in the context of homing behavior, with some of the most striking examples coming from research on desert ants. When foraging in featureless environments, these ants may meander and wind about while moving away from their nests, but they are very proficient at returning directly to the nest without retracing the path that they took on the outward journey (Bühlmann et al., 2011). As path integration pertains to vector algebra, concluding that the desert ant relied on path integration suggests that the ant represented the outward journey from the nest as a series of vectors and then estimated its current location with respect to the nest by summing these vectors (Collett and Collett, 2000). The direct path back is then the inverse of the vector sum.

Finding examples of animal behavior that can be described mathematically is not, by itself, a basis for concluding that the mathematical description corresponds to the internal processing carried out by the animal. However, it is hard to escape this implication with path integration because there is no known way of implementing path integration without also implementing the vector-based computations (Gallistel, 2017).

Among spiders, there is experimental evidence of homing behavior based on path integration from research on an assortment of non-salticid species (e.g., Ortega-Escobar and Ruiz, 2017). Homing by salticids has been demonstrated experimentally (Hoefler and Jakob, 2006), but this has been in the context of relying on landmarks instead of path integration. There is evidence of salticids relying on path integration (Hill, 1979) but, instead of being in the context of homing, this has been in the context of taking detours while pursuing prey.

In the detouring experiments we discussed earlier, the objective was not to look for evidence of path integration, but rather to look for evidence of *Portia* making a plan to access prey that is no longer visible while the plan is being implemented. *Portia*’s behavior in these experiments can be characterized as “navigating,” but with *Portia*’s primary navigational decision being to reach the beginning of the correct path. This might entail *Portia* moving directly away from the location of the prey, and it might mean walking past the beginning of the incorrect path before reaching the beginning of the correct path. However, there was no need for *Portia*’s plan to be inclusive of every twist and turn along the correct path. Once on the path, *Portia* only had to reach the end of that path, with the prey remaining out of view.

In the field, *Portia* often negotiates more complex detouring paths that include multiple branches (Jackson and Wilcox, 1993) and require repeated directional decisions. Although observations from the field might suggest ways in which *Portia* could be used in research more directly related to navigation, including navigation by path integration, these more complex settings for detouring have not been simulated using *Portia*. For this, we can turn to research on salticids that inhabit vegetation and normally target active insects as prey in complex three-dimensional habitats.

In a series of elegant experiments, Hill (1979) demonstrated how salticids from the genus *Phidippus* navigate along paths with side branches. *Phidippus* normally adopts a reconnaissance position on a plant and, after sighting an insect that is inaccessible by a direct path, *Phidippus* takes multiple short detours to reach successive vantage points in the vegetation before arriving close enough to complete the prey-capture sequence. In experiments using artificial plants, Hill demonstrated that *Phidippus* identifies an accessible part of the artificial plant closer to the prey (the “secondary goal”) and then makes a detour to the secondary goal, during which time the prey was moved out of view. Upon arriving at the secondary goal, *Phidippus* then re-orients accurately toward the location of where the prey would have been had it not been moved. The re-orientation data are evidence of *Phidippus* having implemented path integration, based on summing vectors in three dimensions, with respect to the prey’s location as seen from the reconnaissance position on the plant.

Although *Phidippus*’s detours are short compared with *Portia*’s, *Phidippus* takes detours in a setting where additional directional decisions are needed. In other words, after completing a short detour, *Phidippus* can quickly identify the location of the prey from its new vantage point and then take another detour to get closer to the prey’s location. By taking successive short detours based on successive use of path integration, and then re-orienting to the prey’s location, *Phidippus* navigates through the vegetation, a complex physical habitat, to arrive at the primary goal, the prey. This differs from path integration in the context of homing because, in these experiments, path integration was implemented by a test spider with respect to a distant prey individual’s location instead of with respect to the test spider’s own earlier location. *Phidippus* using path integration in the context of navigating to distant prey seems to depend critically on the exceptional capacity for spatial vision supported by salticid eyes.

## *G* and Brain Size

When the notion of larger values for *G* or *g* requiring larger brains approaches the status of an axiom, it becomes unsurprising that vertebrate examples dominate the literature on animal intelligence (Chittka and Niven, 2009; Logan et al., 2018). All the while, there is the inconvenient fact that most animal species are arthropods, and the differences in brain size are enormous when we compare most vertebrates to arthropods. There is also a tendency for vertebrates to have a much slower pace of development and a much longer lifespan than is typical for arthropods. The way vertebrate intelligence is typically discussed may make it seem that, *ceteris paribus*, the expression of intelligence by arthropods can only be negligible. Yet, when we look at examples from spiders and *Portia* in particular, we find capacities that are routinely discussed as examples of intelligence when they are expressed by vertebrates. Moreover, *Portia* is not an isolated aberration. It does not take long to find many comparable examples from research on other arthropods, especially bees and ants (Chittka and Niven, 2009).

When the focus is on vertebrates, the discussion tends to be directed more toward looking for potential advantages gained by having larger brains, but including arthropods in the discussion may shift the discussion more toward looking for potential handicaps or limitations imposed by having minute brains (Eberhard and Wcislo, 2012). Common sense leads us to expect severe limitations more widely than just in the context of intelligence, and some of these other contexts might be more amenable to objective measurement than intelligence currently is for spiders. For example, as an orb-weaving spider’s web is a detailed record of the numerous intricate decisions made when building the web, data acquired from examining webs can be used for comparing the precision with which smaller and larger spiders build their webs. Yet, when detailed comparisons were made, no evidence was found of smaller orb weavers building less precise webs. With this being the case despite orb-weaving spiders varying in body mass by 400,000 times (Eberhard, 2011; Eberhard and Wcislo, 2011), these spiders give us a rather emphatic warning that intuition related to the consequences of small brain size can be misleading (Eberhard and Wcislo, 2012).

We are not proposing that brain size is irrelevant. Envisaging a ceiling on what is possible with respect to intelligent behavior still seems valid (Harland and Jackson, 2004) and it still seems to be a matter of common sense that this ceiling will be lower for arthropods with their minute nervous systems and higher for vertebrates with their enormously larger nervous systems. However, if addressing Macphail’s null hypothesis is of interest, then arthropod-vertebrate comparisons might be a good place to start. Discussing the null hypothesis only in the context of vertebrate-to-vertebrate comparisons seems excessively arbitrary. It seems to us that, when the goal is to identify qualitative and quantitative differences in intelligence, the context should be inclusive of all animals that express capacities pertaining to intelligence, irrespective of whether they are vertebrates.

## Author Contributions

All authors contributed to the article and approved the submitted version.

### Conflict of Interest

The authors declare that the research was conducted in the absence of any commercial or financial relationships that could be construed as a potential conflict of interest.
